# Some New Local Anæsthetics

**Published:** 1907-01-05

**Authors:** 


### SOME NEW LOCAL ANÆSTHETICS.

### Alypin.

THIS drug, one of a group of local anæsthetics analogous in their mode of application to cocaine, is the hydrochlorate of benzoyl 1-3 tetramethyl-diamino 2 ethyl-isopropylic alcohol. It is a white crystalline powder, very soluble in water, and not precipitated in weak alkaline solutions, nor altered by boiling.

*Mucous Membranes.*—A 10 per cent. solution in water has been successfully used for local application in operations about the throat and nose.

*Minor Operations and Dental Surgery.*—For this work subcutaneous injections of 0.5 to 1.5 c.c. of a 2 or 3 per cent. solution are recommended, but it is pointed out that there may be considerable irritation and destruction of tissue at the site of injection with even so dilute a solution as 5 per cent.

*Spinal Anæsthesia* has been satisfactorily produced without unpleasant symptoms by the intra-spinal injection in the lumbar region of a 3 per cent. solution to the amount of 5 c.c., permitting of the majority of operations below the level of the umbilicus.

*Ophthalmic Surgery.*—In this department particularly alypin is found to provide a non-irritating, rapid, conjunctival and corneal anæsthetic; the strength advocated for instillation in the eye is from 2 to 10 per cent. It does not affect the cornea and has no effect on the pupil or accommodation.

### Novocain.

Novocain is the monochlorhydrate of para-amino-benzoyl-diethyl-amino-ethynol, and is freely soluble in water (1 in 1) and in alcohol (1 in 30), the aqueous solution having the great advantage, that it can be boiled without decomposing. Even in concentrated solutions its injection does not cause local irritation and its toxicity is slight.

*Mucous Membranes.*—It is frequently employed for operations on mucous surfaces, particularly as a prelude to the intra-urethral application of silver nitrate in urethritis, in 5 to 10, or even 20 per cent., solutions, and may be combined with 1 in 1,000 suprarenal solution.

*Minor Operations.*—For subcutaneous injection solutions of 0.25 to 2 per cent. may be used without any ill effects from absorption or local irritation.

*Spinal Anæsthesia.*—As an intraspinal injection 2 or 3 c.c. of 5 per cent., or 1.25 to 1.8 c.c. of a 10 per cent. solution, combined with suprarenal solution, have been found very satisfactory and attended by no disagreeable symptoms.

*Ophthalmic Surgery.*—Novocain has the great advantage when instilled into the conjunctiva (2 to 10 per cent. solution) of causing rapid anæsthesia without mydriasis, and it may be combined in this case also with suprarenal solution, thus presenting an almost ideal anæsthetic for ophthalmic work.

*Dental Surgery.*—Here a solution of 1 or 2 per cent. is usually found sufficient, and the effects very satisfactory.

### Stovain.

The hydrochlorate of dimethylamino-benzoyl-dimethyl-ethyl carbinol is another of the several recently discovered local anæsthetics. Although freely soluble in water the solution has the disadvantage of being acid in reaction, and therefore easily precipitated when brought into contact with weak alkaline fluids such as the body tissues contain. It has certain toxic effects: violent headache, giddiness, nausea, and vomiting, but these seem to be chiefly observed in lumbar anæsthesia in pregnant women, to whom Chartier has administered the drug by spinal injection to stimulate uterine contraction while diminishing its painfulness. These labour-inducing properties should be borne in mind.

*Minor Surgery.*—Local injections for surgical operations may be made with 0.5 to 1 per cent. solutions in doses not exceeding 2 grains of stovain: upon intact mucous membranes it is practically inert.

*Spinal Anæsthesia.*—In this, however, it plays a most important part; the injection takes the form of a 10 per cent. solution, of which 3 to 7 c.c. will in five to eight minutes induce an anæsthesia lasting from one to eight hours—a longer period than can be secured with any other intraspinal injection.

*In Ophthalmic Surgery* stovain proves useful for subcutaneous or subconjunctival injection, but not so for mere instillation, where it is quite ineffective.

*Dental Operations* may be painlessly carried out by the injection of 2 c.c. of a 0.75 or 1 per cent. solution into the gums, but it is not recommended for difficult or forcible extractions. Stovain has many incompatibles—notably alkalies, many mercurial compounds, iodine, and the iodides.

### Morphia-Scopolamine.

To a different chemical category, but with a similar clinical application, belongs the intra-spinal injection of scopolamine hydrobromide combined with morphia for inducing anæsthesia. The method undoubtedly has many advantages, but its drawbacks are the length of time which elapses between the injection and the establishment of anæsthesia ($1\frac{1}{2}$-2 hours), and it is not free from uncertainty and danger, but in many thousands of administrations the mishaps have been few and far between.

The solutions should always be freshly prepared and carefully sterilised, small repeated doses should be given (scopolamine gr. $\frac{1}{120}$, morphia gr. $\frac{1}{40}$), beginning an hour before the intended operation, one to three injections perhaps being required.

The method is dangerous in children and in adults with heart or kidney disease; in addition to surgical operations the spinal injections have proved beneficial in violent spasmodic contractions of the uterus during labour and in nervous women, also for cystoscopic and urethral examinations. It is recommended by some authorities for the pains of tabes and in cases of inoperable tumours, while it has a marked influence upon the symptoms of paralysis agitans, although, unfortunately, its effect lasts only some twelve hours.

**Tropacocain.**

The hydrochlorate of tropacocain appears so far to hold its own in the field of spinal anæsthesia.

It is almost completely innocuous even in large doses and in concentrated solution: debility, old age, and arterio-sclerosis do not contra-indicate its use, although it is generally agreed that it should not be employed in patients who have not reached the age of puberty.

*Spinal Anæsthesia.*—The dosage for spinal injection is 0.05 to 0.08 grain in 2 per cent. solution, gradually increasing if necessary until complete anæsthesia ensues. The method is particularly applicable for operations on the lower limbs and external genitals, including the vagina and uterus, provided the peritoneum is not opened. With extensive pelvic adhesions it is not advisable to make use of spinal anæsthesia, and the Trendelenburg position may involve the danger of the tropacocain spreading into the upper parts of the spinal cord. Decrepit subjects whose condition forbids the use of general anæsthetics by the inhalation method bear the spinal injection well.

*Dental Surgery.*—In dental practice very good results have been obtained by local injection of tropacocain.

*Veterinary Practice.*—Both experimentally and in actual surgical operations on animals tropacocain has been successfully employed by the intraspinal method.

